# Prevalence of Physical Violence in the Medical-Forensic Approach in the Years 2015–2020 in City and Neighboring Municipalities: Perspectives from Poland—Poznań Study

**DOI:** 10.3390/ijerph20042922

**Published:** 2023-02-07

**Authors:** Szymon Rzepczyk, Klaudia Dolińska-Kaczmarek, Bartosz Burchardt, Dagmara Skowrońska, Przemysław Hałasiński, Aleksandra Bielecka, Klaudia Koniarek, Czesław Żaba

**Affiliations:** Forensic Medicine Departament, Poznan University of Medical Sciences, ul. Rokietnicka 10, 60-806 Poznan, Poland

**Keywords:** violence, statistics, crime prevention and intervention, safety, physical abuse

## Abstract

Forensic medical opinions serve the appropriate classification of a crime against health. Violence, a multifaceted phenomenon, requires forensic medical examination in the case of causing damage to health. Due to the effects caused by the perpetrator, the damage to health is divided into severe, medium, and light. This study analyzed 7689 incidents of violence from 2015–2020, taking place in the area subordinate to the Provincial Police Headquarters in Poznań, based on anonymized documentation of forensic medical examinations performed at the Department of Forensic Medicine in Poznań at the request of the Police and privately. The analysis took into account: units ordering the test, type of exposure, medical help, sex and age of the victim, places of the incident, classification and localization of injury, manner of impact, attitude of the perpetrator to the victim, profession of the victim, gender of the perpetrator, and remarks. In Poland, statistics on violence victims are underestimated, resulting from the low reporting of crimes committed to law enforcement authorities. There is a need for programs to educate the perpetrator of violence on methods of conflict resolution and programs to prevent violence, covering events taking place in public spaces.

## 1. Introduction

Physical violence is a common phenomenon that is a complex social problem. Poorly adapted legal systems, low levels of education in this area, stereotypes, or the so-called “victim blaming” are just some of the issues that victims of violence have to face [1].

### 1.1. Forensic Examination

The element that allows the procedural authority to classify a crime against health is forensic medical opinions [2]. In light of the provisions described, a forensic medical examination is a basis for issuing a competent statement. It examines the victim with a detailed description of the injuries and their location [3]. Based on the analysis of visible injuries, the expert issues a medical opinion containing conclusions on the severity of the injury and damage to health and answers to additional questions asked by the investigating authority [3,4]. The result of the examination and the opinion shall be presented as a report to be submitted to the managing authority. Proper opinion preparation requires impartial specialist knowledge of the mechanisms of injury and their effects, linking them into cause-and-effect sequences, and expert knowledge of legal provisions and their application rules [5]. Particular attention should be paid to expert opinions on the conditions of exposure to damage to health or loss of life, where the material to be analyzed does not have to be only measurable biological effects but the assessment of the circumstances of the event with its potential effects [6].

### 1.2. Legal Aspects—Polish Perspective

Violence is a multifaceted phenomenon that can be discussed in a social, psychological, or legal context. A particular type of violence is domestic violence. Violence can take many forms, one of which is causing bodily harm. It is a broad concept because it includes violations of the functions of the body’s organs and disorders of various types and degrees. The legislator adopted a division into three kinds of damage to health due to the effects caused by the perpetrator—severe, medium, and light. Each of them is a particular, intrinsic type of criminal activity. They do not, therefore, form a primary, privileged, or suitable kind for each other [7].

Severe damage to health in the primary type is regulated in Article 156 §1 of the Penal Code, under which the legislator has separated two groups of behaviors. The first is the deprivation of sight, hearing, speech, and the ability to procreate. The second one included causing another severe disability, a serious incurable or long-term illness, an actual life-threatening illness, a permanent mental illness, total or significant incapacity for work in the profession, or permanent, considerable disfigurement or deformity of the body [8]. The structure of the provision is therefore based on the indication of the effect defined by the legislator as severe damage to health and a casuistic specification of its various forms [9].

The legislature divided the category of ‘other’ into that specified in Article 156 §1 of the Penal Code into two groups according to the criterion of their duration. The effect of a violation of bodily function or health disorder lasting less than seven days was classified as mild (Article 157 §2 of the Penal Code), while the effect lasting more than seven days as medium (Article 157 §1 of the Penal Code) [9]. Both of the above offenses can be committed both intentionally and unintentionally.

The relationship between the victim and the perpetrator of the offense is irrelevant to the offense’s actual occurrence or the offender’s treatment by the Court. However, it is relevant to the mode in which the crime will be prosecuted, as specified in detail in Article 12 of the Code of Criminal Procedure [10].

These goods are so essential that, in the legislator’s opinion, their criminal law protection is necessary not only at the stage of their violation but also at the stage of exposing them to danger. This protection is implemented using Article 160 of the Penal Code, penalizing human exposure to danger. The constituent elements of this prohibited act may be implemented in one of three ways—by bringing a threat, by significantly increasing it (Article 160 §1 of the Penal Code), and also—in the case of persons who are obliged to care for a person exposed to danger (Article 160 §2 of the Penal Code)—by not causing its resignation or reduction [11]. In the Polish legal system, a two-instance judiciary is standard, with the possibility of cassation appeal to the Supreme Court supervising the operation of the courts. The functioning of the Polish justice system within the principles of the established law system enables a significant differentiation of jurisprudence between courts thanks to the discretionary freedom of the judiciary. This differentiation is particularly noticeable in the case of the provision discussed above. However, an expert opinion is necessary for correctly classifying the act by the managing authority having such powers. However, the expert does not formally have the right to determine the act’s type, and the court may not agree with his opinion.

A distinction must be made between bodily harm offenses that do not result in a breach of organ function or health disorder. Article 217 of the Penal Code provides for criminal liability for an act violating bodily integrity, constituting an attack on the right of every person to maintain personal integrity. It is also essential that criminal liability under Article 217 of the Penal Code is not conditioned by the victim’s awareness of violating his bodily integrity [12].

## 2. Materials and Methods

The analysis covered 7689 incidents of violence from 2015–2020 taking place in the area subordinate to the Provincial Police Headquarters in Poznań. The study was based on anonymized documentation of forensic medical examinations performed at the Department of Forensic Medicine in Poznań at the request of the Police and private (80.3% and 19.7% of all tests performed, respectively). Descriptive statistics were carried out in the following categories: number of incidents per year and per commissioning entity, victim’s exposure to the health damage, the use of medical help, victim’s age and sex, incident location, type of injury, body location of the injury, method of impact, offender-victim ratio, the profession of the victim, gender of the perpetrator, and additional remarks.

Additional remarks include information the authors found interesting and important for future studies that were not matched to the main categories, such as incidents committed by more than one perpetrator, juvenile perpetrators, pregnant victims, or victims’ ethnicity.

The results are presented as a percentage.

Descriptive statistics were used: mean, median, standard deviation (SD), first and third-quartile values (IQR), and range.

The significance level was *p* = 0.05, but results relevant to the levels *p* = 0.01 and *p* = 0.001 were also indicated. Any *p*-values that showed statistically significant results were highlighted in bold. For *p* < 0.0001, the notation *p* < 0.001 has always been used.

All calculations and graphs were made using the statistical package R v. 4.0.2.

## 3. Results

Based on the obtained data from the victims during the obduction, 8893 perpetrators were recorded, of which men accounted for the vast majority (84.3%). Unknown perpetrators were the most common (40.0%). Following the analysis, 7689 victims were registered, representing men (59.5%) and women (40.4%). The age of the examined persons was between 1 and 94, with an average age of about 37 and a median of 35. The highest incidents were recorded in public places (52.9%). Physical workers and students were the most commonly exposed to violent actions. The most significant number of injuries were caused by fist strikes (26.9%). The types of injuries in the majority were bruises and contusions (45.1%), with the most prevalent injury site being the head and neck area (30.6%).

**Figure 1:**
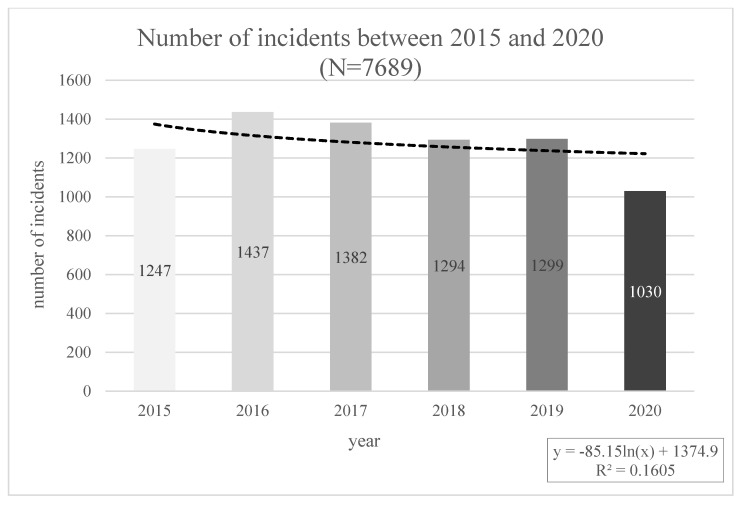
The number of incidents between 2015 and 2020.

The frequency of recorded incidents between 2015 and 2020 is presented in Figure 1. The highest number of forensic examinations was conducted in 2016. (*n* = 1437) and the fewest in 2020 (*n* = 1030). The reason for the low number of examinations conducted in 2020 might result from the start of the COVID-19 pandemic, the restrictions and limitations introduced, and thus the lower frequency of reporting by victims of incidents, as well as reducing the activities of Forensic Medicine Department in Poznan and the related cessation of examinations from March 22 to May 15. Disregarding the exceptional year 2020, due to the events mentioned above, the number of incidents oscillates at a similar level during the period under review.

The most significant number of examinations were performed based on private orders (*n* = 1516; 19.7%) and orders from Police Commissariats located in the city of Poznań, i.e., Grunwald (*n* = 943; 12.3%), New Town (*n* = 831; 10.8%) and Old Town (*n* = 792; 10.3%). The smallest number of orders came from the Konin City Police Station and the Czerniejewo Police Station (Table 1).

Moreover, out of the forensic examination, 6607 (85.9%) failed to comply with the criteria for violating bodily functions or health disorders lasting more than seven days (Table 2). In 23 cases, it was impossible to determine this parameter, which may have resulted, for example, from the victim’s failure to appear for a reassessment of injuries, despite recommendations.

A total of 4371 cases (56.8%) involved visits to the ED/Emergency Room or the arrival of the Emergency Medical Service (EMS) (Table 3).

However, the injured required hospitalization in only 4% of the analyzed cases. Hospitalization requires admitting the injured person to a hospital ward and performing surgical procedures due to the injuries suffered.

The injuries were sustained by both men (59.5%) and women (40.4%), and in only two cases was it impossible to determine the sex (Table 4).

The victims’ mean age was approximately 37 years and the median 35 years, with a range of participants from 1 to 94 years old (Table 5).

Frequently, incidents occurred in public places (52.9%), further in private areas (39.1%), and the fewest events were recorded at the workplace (8.0%). Public places refer to stairwells, allotments, gates, parking lots, schools, stores, and other areas of public use (Table 6).

Furthermore, amid all recorded injury types (*n* = 12,505), the most common injuries found in reporting victims of violence were mild injuries in the form of bruises and contusions (*n* = 5641; 45.1%), surface abrasions, redness, and discoloration (*n* = 3675; 29.4%) (Table 7).

Additional observed injury types occurring at a rate of less than 1% were joint sprains and twists, concussions, muscle and tendon injuries, thermal burns, lacerates, stab and gunshot wounds, hair loss, amputations, ear injuries, internal injuries, chemical injuries, eye injuries, and frostbite. No injuries were reported in 646 cases.

Of the total injury sites (*n* = 15,537), the head and neck (*n* = 4759; 30.6%) and upper limb were the most affected: forearm and hand injuries accounted for 17.0%, and injuries in the arm and shoulder girdle were 16.0% (Table 8). Less frequent injuries were observed in the case of lower limbs, as lower leg and foot injuries accounted for 9.9% while the thigh and hip joints for 9.0%.

In 641 cases, no visible injuries were reported. The fewest reported injuries were in the genital region (*n* = 16; 0.1%) and internal injuries (*n* = 2; 0.01%).

Table 9 summarizes the perpetrator’s methods of inflicting injuries, with frequencies recorded at a total of 13,171. Most commonly, the perpetrator was punched with a fist (*n* = 3543; 26.9%) and jerked and pushed and used a firm grip or twisted and dragged by the hair (*n* = 3248; 24.7%).

The objects most often used to cause injury were complex and blunt-edged objects (*n* = 1050); next sharp-edged things such as a knife or axe (*n* = 170), and a minor percentage were the use of leather belts, cables, and rope (*n* = 24) or firearms (*n* = 12).

Table 10 shows data on the perpetrator’s relationship with the victim. However, in as many as 40.0% of cases, the perpetrator was unknown to the victim, and in 32.0% of the perpetrator was known to the victim, the relationship was not specified. In addition, a high incidence of violence perpetrated against victims by a spouse (10.0% of perpetrators) or current partner (4.2%) was observed.

In a small percentage of no more than 1%, injuries were caused by an animal, a parent’s partner, medical personnel, or workplace equipment.

**Figure 2:**
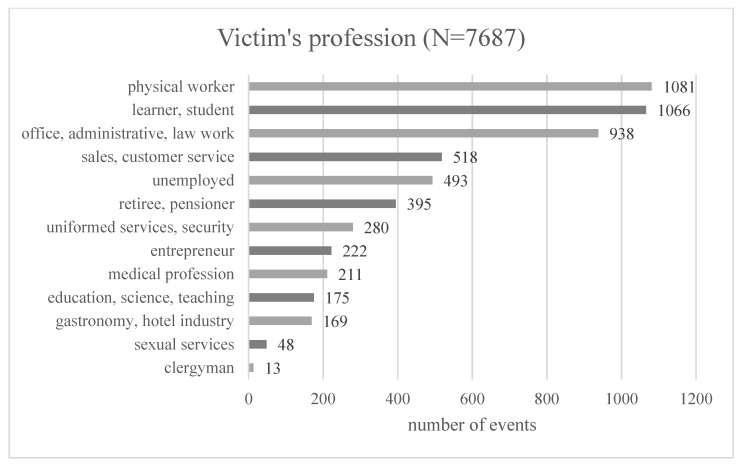
Victim’s occupation.

The professional profile of victims reporting for medical examinations was also analyzed. Data are shown in Figure 2. Regarding the 5609 occupations reported (unknown occupations were not included), the most significant proportion were physical workers (19.3%) and students (19%). Subsequently, office workers had the highest number of injuries, accounting for 16.7% of victims. The unemployed were victims in 8.8% of the discussed cases.

In analyzing the documents from the examinations, additional criteria were taken into account, such as the involvement of more than one perpetrator, offenses under Article 217 of the penal code, unconsciousness of the victim, the action of a minor perpetrator, the origin of the victim other than Polish (division into post-Soviet, EU, non-EU and post-Soviet countries) as well as self-harm and pregnant victims. In as many as 1204 events, two or more perpetrators committed acts of violence.

**Figure 3:**
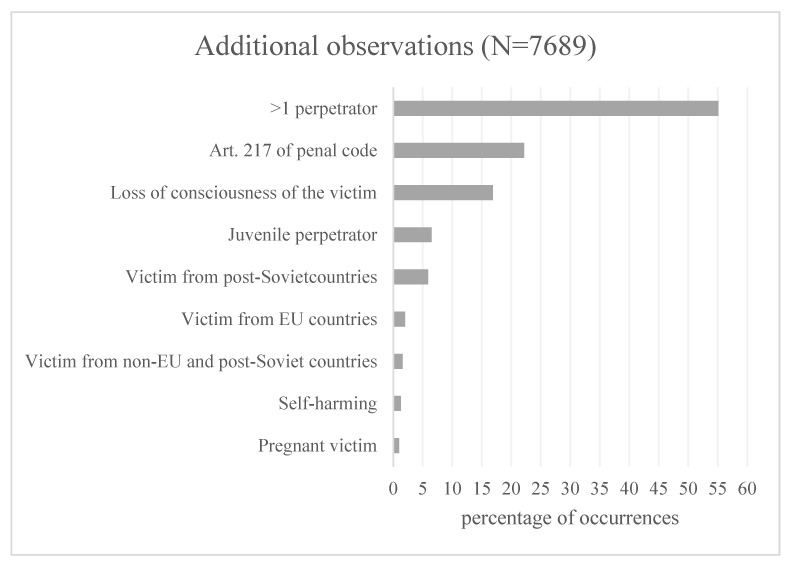
Additional observations.

The victims included 207 people of non-Polish origin, of whom 128 were from post-Soviet countries (e.g., Ukraine, Belarus) and 43 from countries belonging to the European Union. In 28 cases, the injuries were self-inflicted. All of the additional observations are summarized in Figure 3.

**Figure 4:**
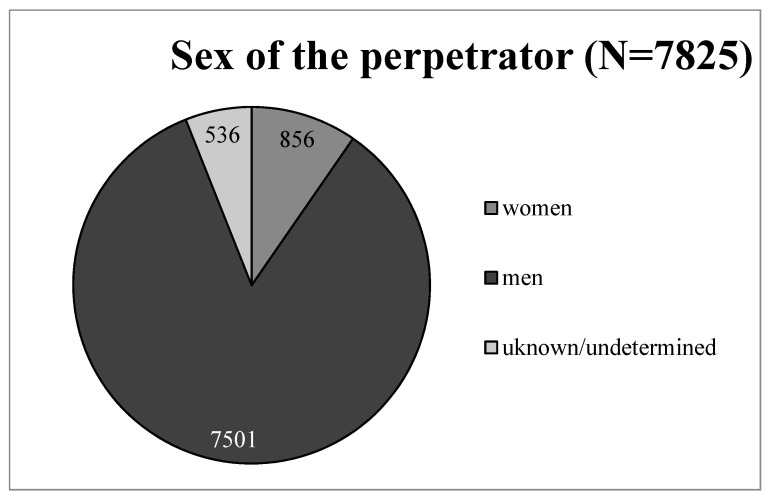
The distribution of perpetrators by sex.

As reported by the victims, the perpetrators were most often men (84.3%), and only 9.6% of the noted cases were women (Figure 4).

## 4. Discussion

### 4.1. Comparison of Analysis Data with Publicly Available Data

The Police Headquarters keeps statistics on domestic violence in Poland. In Poland, statistical data are compiled based on the number of “Blue Cards” issued [13]. A “Blue Card” means the commencement of legal proceedings for suspected domestic violence [14]. Police statistics include the number of completed “Blue Card” forms; the number of people suspected of being affected by violence; the number of people suspected of domestic violence; the number of people suspected of domestic violence under the influence of alcohol [13]. These data illustrate the scale of domestic violence in Poland. Between 2015 and 2020, the number of Blue Cards completed was 444,755, while the number of people suspected of being affected by violence was 543,559, of which women accounted for about 73%. European statistics on domestic violence are based on a survey conducted by the European Union Agency for Fundamental Rights in 2014, which involved 42,000 women aged 18–74 [15]. Among other things, it found that about 33% of women have experienced physical or sexual violence after age 15, including about 11% of sexual violence during their lifetime. Partners of victims committed 78% of acts of physical violence over 15. The most common forms of physical violence were: pushing or shaking, slapping or grasping, or pulling women by the hair. In turn, our study covered 7689 incidents of violence in the Provincial Police Headquarters in Poznań. Women and men were violence victims (40.4% and 59.5%, respectively). The median age of victims of violence was 35 years. The most common perpetrators are men—82.8% and the most frequently observed way of inflicting damage are using fists (26.9%) and jerking, pushing, pulling the hair, twisting, and using a firm grip (24.7%). Sexual violence accounts for 1.2% of the way bodily harm is performed. The low reporting of sexual violence in Poland is due to the leniency of the justice system and the difficulty in proving that the sexual act occurred as a result of violence and that the resistance offered by the victim was strong enough [16]. The highest percentage of the offender’s relationship with the victim refers to the group of unknown and known people, followed by the group of close people (22.3%). Among close relatives, spouses are the most common perpetrators (45%).

### 4.2. Violence Prevention Programs in Poland

Presently, there are several programs to prevent violence in Poland. One that functioned in the years covered by this research (2015–2020) was the National Program for Prevention of Violence in the Family for 2014–2020 and the Government Program for 2014–2016 “Safe and Friendly School” [17,18]. In addition to nationwide programs, there are also programs covering smaller local government units, i.e., voivodeships, municipalities, or counties (for instance, communal violence prevention programs). They include social campaigns, training, and legal and psychological assistance for victims of violence. According to the authors, it is insufficient, as government actions mainly focus on violence within families and schools. Based on the analysis, the violence’s most frequent perpetrators were people unrelated to the victim—for this reason, the “Blue Card Program” alone is inadequate. Adults are the most common perpetrators of violence—juvenile perpetrators accounted for only 142 cases in the total statistics. Although the authors do not negate the importance of anti-violence campaigns in schools, they point out that without efforts beyond domestic and workplace violence, it will be unattainable to achieve real improvements.

Moreover, another critical aspect is the absence of programs aimed at perpetrators of violence, particularly in psychoeducation, including ways to resolve conflicts and control emotions.

### 4.3. Programs to Prevent Violence in the Provincial Police Headquarters in Poznań

The main program for counteracting violence in Provincial Police Headquarters in Poznań is the National Program for Preventing Domestic Violence dealing with implementing the “Blue Card” procedure. According to reports from the Voivodship Office, 20,476 people fell victim to domestic violence in 2019, translating into 8922 families covered by the “Blue Card” procedure [19]. These numbers have been steadily increasing since 2015. In response to the growing number of victims and to enable them to access assistance, the number of Working Groups appointed by Interdisciplinary Teams (by the provision of Article 9a (10) of the Act mentioned above on counteracting domestic violence) to solve problems related to the occurrence of domestic violence in individual cases has also increased. In 2015, 5190 such groups were established, and in 2020 already, 6788 [20].

The disparity between the number of victims of domestic violence and data collected for this study may be explained by the fact that only about 8% of victims notify the prosecutor’s office or other law enforcement authorities. For example, in 2019, 8922 Blue Card procedures were conducted. Still, Interdisciplinary teams submitted 715 notifications to law enforcement agencies (police, prosecutor’s office) on suspicion of committing a crime in connection with the use of domestic violence [19].

It is also worth noting that smaller-scale programs focus mainly on disseminating information on violence, i.e., how to recognize and react to it and how the victim can attain the help they need. Particularly noteworthy is the Wielkopolska Violence Prevention Program organized by the Regional Center for Social Policy in Poznań, which aims to deepen the awareness of Wielkopolska residents, help victims, and work with the perpetrator of violence to break the cycle of violence [21]. As part of their activities, they organize, among others, several lectures and educational meetings, training for teachers and pedagogues, and managing psychological duties for victims of violence [22].

### 4.4. Compare Data on Interpersonal Violence Overall in Europe and Worldwide

There are no examples of similar studies in the literature. Most of the available data focus on domestic violence and violence against minors. This leads to the conclusion that interpersonal violence is marginalized in some way and rarely becomes the object of scientific analysis. In the analysis presented in the above work, it can be seen that more than half of the incidents (52.9%, *n* = 7689) took place in a public place, and 4 out of 10 perpetrators were not personally known to the victims. Jud et al. (2023) conducted a study on the German population, receiving 2503 responses in surveys sent to 5668 households for speech. The resulting distribution corresponded to the German population over 14 years of age. A total of 57.6% of female participants reported having been victimized by intimate partners during their lifespan; male participants affirmed having been victimized at a rate of 50.8% [23]. Tingne et al. (2014) presented an analysis of violent injuries from the perspective of hospital emergency department workers in India. They analyzed 813 reports occurring in one year. The vast majority of injuries were blunt injuries to the head and neck area. The group most vulnerable to violence were young men, usually attacked by unknown men in public. On the other hand, women were more likely to be victims of domestic violence [24]. Similar results were presented by scientists from the UK—Sivarajasingam et al. (2017) [25] and Olding et al. (2019) [26]. Particularly noteworthy is the analysis of the phenomenon of domestic violence in six European countries, in which Costa et al. (2015) proved that both men and women are equally likely to become victims and perpetrators of domestic violence [27,28]. However, it is worth noting that men are increasingly admitting to being victims of domestic violence, which is probably related to cultural changes and breaking taboos [29,30,31]. A similar analysis by Lindert et al. (2011) of seven European cities found that women are more likely to be victims of violence in the older population (60–84 years) but significantly more likely to be psychological and economic rather than physical [32]. The cited research forces the search for solutions to prevent violence and poses new challenges for authorities, law enforcement services, and healthcare workers. Awareness of the scale of the problem, the most common types and locations of injuries, and the debunking of the myth that women are victims and men are perpetrators of violence is crucial for the proper recognition of victims and comprehensive assistance and education activities [33,34]. An important part of the help chain is access to the victim’s medical history. It is important that both general practitioners and hospital emergency department staff recognize and record characteristic injuries in order to compare the injuries with the history given by both the perpetrator and the victim of violence in the future [35,36,37,38].

## 5. Conclusions

The authors believe Poland has insufficient systemic solutions to facilitate reporting crimes. In many environments, violence is perceived through stereotypes, making it difficult to obtain real support from relatives and law enforcement agencies. The measures taken by the government and local authorities, combined with the Polish legal system and incident reporting procedures, are insufficient to help the victim easily and comfortably. Bottom-up initiatives led by private actors compensate for some gaps, but more is needed.

According to the authors, it is necessary to develop systemic legal solutions and increase expenditure on educational campaigns to counteract violence. It is also important to train medical staff, in particular family doctors, employees of emergency rooms, nurses, and community midwives, to alert them to the signs of violence visible in the patient and to enable a quick response.

## Figures and Tables

**Table 1:** The number of incidents by the commissioning entity.

The Commissioning Entity	N (7689)	%
Private orders	1516	19.7
Police Station Poznań Grunwald	943	12.3
Police Station Poznań Nowe Miasto	831	10.8
Police Station Poznan Stare Miasto	792	10.3
Police Station Poznań Jeżyce	631	8.2
Police Station Poznań Północ	565	7.3
Police Station Poznań Wilda	504	6.6
Municipal Police Station in Poznan	143	1.9
Provincial Police Headquarters in Poznan	22	0.3
Military Police	3	0.0
Police Station in Swarzedz	251	3.3
Police Station in Tarnow Podgórny	233	3.0
Police Station in Luboń	155	2.0
Others	1100	14.3

**Table 2:** Categorization of incidents by victim exposure.

Parameter	Damage to Health Lasting More than Seven Days (art. 157 of the Penal Code)			Exposure to Medium and Severe Damage to Health			
	Yes	No	Unknown	art. 157 §1 of the Penal Code	art. 156/160 of the Penal Code	No	Unknown
N (7689)	1059	6607	23	497	1064	6083	45
%	13.8	85.9	0.3	6.5	13.8	79.1	0.6

**Table 3:** Division by medical exigencies.

Parameter	Hospitalization			Emergency Room		
	Yes	No	Unknown	Yes	No	Unknown
N (7689)	311	7376	2	4371	3315	3
%	4	95.9	0.03	56.8	43.1	0.04

**Table 4:** Incident distribution by victim sex.

Parameter	Sex of the Victim		
	Woman	Man	Unknown
N (7689)	3110	4577	2

**Table 5:** Statistical results of the age of victims.

Victim’s Age [Years]			
N	Mean (SD)	Median (IQR)	Range
7684	36.73 (15.66)	35 (25–46)	1–94

**Table 6:** Division of incidents by location.

Parameter	Place of Incident		
	Public	Private (Flat/Apartment/House)	Work
N (7689)	4068	3007	614
%	52.9	39.1	8.0

**Table 7:** Incident classification by type of injury.

Injury Type	N (12,505)	%
Bruise, contusion	5641	45.1
Surface abrasion, redness, discoloration	3675	29.4
Contused wound	703	5.6
Bone fracture	679	5.4
No injuries	641	5.1
Dental injuries	372	3.0
Cut wound	261	2.1
Others	533	4.3

**Table 8:** Locations of Injury (*n* = 15,537).

Location of Injury	N	%
Head and neck	4759	30.6
Abdomen	416	2.7
Leg	1535	9.9
Forearm	2645	17.0
Tigh	1391	9.0
Thorax	909	5.9
Back and gluteus regions	738	4.7
Genital regions	16	0.1
Internal injuries	2	0.0
No injuries	641	4.1

**Table 9:** Way of inflicting damage.

Variable	Parameter	N (13,171)	%
Way of inflicting damage	Punching with a fist	3543	26.9
	Jerking, pushing, pulling hair, twisting, firm grip	3248	24.7
	Kicking	1853	14.1
	Hard, blunt-edged object	1050	8.0
	Open hand	913	6.9
	Strangling	588	4.5
	Unknown mechanism	394	3.0
	Biting	195	1.5
	Irritant substances (e.g., pepper spray)	194	1.5
	Head impact	191	1.5
	Sharp-edged (e.g., knife, axe)	170	1.3
	Sexual violence	164	1.2
	Others	668	5.1

**Table 10:** The relationship between the perpetrator and the victim.

The Relationship between the Perpetrator and the Victim	N (7758)	%
Unknown	3103	40.0
Known (unspecified)	2480	32.0
Spouse	779	10.0
Partner	328	4.2
Another close family member	236	3.0
Ex-partner	184	2.4
Police officer, Municipal Police, security guard	179	2.3
Parent	170	2.2
Further family	158	2.0
Other	141	1.8

## Data Availability

All data supporting reported results can be found in the archive of the Forensic Medicine Department of Poznan University of Medical Sciences.
